# Transition Behavior in Blended Material Large Format Additive Manufacturing

**DOI:** 10.3390/polym18020178

**Published:** 2026-01-08

**Authors:** James Brackett, Elijah Charles, Matthew Charles, Ethan Strickland, Nina Bhat, Tyler Smith, Vlastimil Kunc, Chad Duty

**Affiliations:** 1 The Bredesen Center for Interdisciplinary Research, University of Tennessee, Knoxville, TN 37996, USA; cduty@utk.edu; 2 Department of Mechanical and Aerospace Engineering, University of Tennessee, Knoxville, TN 37996, USA; 3 Manufacturing Science Division, Oak Ridge National Laboratory, Oak Ridge, TN 37830, USA; smithtc@ornl.gov (T.S.); kuncv@ornl.gov (V.K.)

**Keywords:** Additive Manufacturing, thermoplastic composites, large-format, multi-material

## Abstract

Large-Format Additive Manufacturing (LFAM) offers the ability to 3D print composites at multi-meter scale and high throughput by utilizing a screw-based extrusion system that is compatible with pelletized feedstock. As such, LFAM systems like the Big Area Additive Manufacturing (BAAM) system provide a pathway for incorporating AM techniques into industry-scale production. Despite significant growth in LFAM techniques and usage in recent years, typical Multi-Material (MM) techniques induce weak points at discrete material boundaries and encounter a higher frequency of delamination failures. A novel dual-hopper configuration was developed for the BAAM platform to enable in situ switching between material feedstocks that creates a graded transition region in the printed part. This research studied the influence of extrusion screw speed, component design, transition direction, and material viscosity on the transition behavior. Material transitions were monitored using compositional analysis as a function of extruded volume and modeled using a standard Weibull cumulative distribution function (CDF). Screw speed had a negligible influence on transition behavior, but averaging the Weibull CDF parameters of transitions printed using the same configurations demonstrated that designs intended to improve mixing increased the size of the blended material region. Further investigation showed that the relative difference and change in complex viscosity influenced the size of the blended region. These results indicate that tunable properties and material transitions can be achieved through selection and modification of composite feedstocks and their complex viscosities.

## 1. Introduction

Additive Manufacturing (AM) has attracted interest for its ability to construct complex geometries typically incompatible with traditional manufacturing methods, which has led to its usage in automotive, aerospace, and biomedical applications [1,2,3,4,5]. Although the type utilized varies with the intended application, AM techniques are expected to be a critical component of the Industry 4.0 revolution [6,7,8]. For thermoplastics and their composites, extrusion-based AM processes have been limited by low deposition rates and smaller build volumes [9]. To address this limitation, Large-Format Additive Manufacturing (LFAM) techniques have been developed that retain the advantages of extrusion-based AM processes while enabling industry-scale production of thermoplastic composite structures [10,11,12,13,14,15,16,17].

However, like many AM sub-fields, LFAM systems have had difficulty printing Multi-Material (MM) structures [18,19,20], leading to the development of Multi-Material Additive Manufacturing (MMAM) [21,22,23,24,25,26,27,28,29,30]. Due to the layer-by-layer nature of AM, fully capable MMAM systems, defined by the ability to utilize more than one material without pre-mixing, pre-compositing, or non-AM post processing treatments [23], would inherently be capable of tailoring structural properties through precise control of material placement during the design and printing process. Although there are many types of MMAM systems currently available, bonding dissimilar materials is a recurring challenge, frequently resulting in structural weak points [28,31,32]. Despite the increased size and build volumes, LFAM systems have also exhibited similar vulnerabilities to delamination failure in MM structures [18].

Functionally Graded Material (FGM) construction—a common method for improving bonding between dissimilar materials by varying material composition, structure, or density in one or more spatial directions [22,33]—has two broad classifications: stepwise and continuous. Stepwise FGM comprise three or more homogenous regions, separated by discrete boundaries, and often place intermediate blends in the “middle” regions [22]. This approach has successfully improved fracture toughness [34], durability [24], and other properties [30] by increasing the number of intermittent regions, or steps, in the stepwise FGM. Characterized by a continuous change in composition, structure, or density in one or more spatial directions [22,33], continuous FGMs offer a unique advantage over their stepwise counterparts by avoiding the presence of discrete boundaries altogether. This advantage has been demonstrated using linear and sigmoid gradients [34], the natural material response of pressure variation [35], and numerous other approaches [3,21,36]. Despite these successes, recent reviews still identify the continued development of MMAM capabilities and FGM construction as crucial to the success of AM in many industries, including dentistry [5], 4D printing [37], construction [18], and light-weighting applications [38].

Developed by Cincinnati Incorporated in cooperation with Oak Ridge National Laboratory, the Big Area Additive Manufacturing (BAAM) system’s dual-hopper design enables automated, in situ material switching by seamlessly alternating between hoppers to control which is supplying material to the extrusion system [27]. This step-change in feedstock creates a compositional gradient similar to those seen in continuous FGMs, leading to three distinct regions [39,40,41,42]. Previous investigations have shown that transition behavior was influenced by screw rotational speed, screw design geometry, and the direction of the material transition (A to B vs. B to A) [39,42].

To objectively compare material transitions printed under a variety of conditions, this study presents a novel framework for analyzing and comparing the printed transitions that ensured accurate evaluation of the contributing factors. Using this framework, the influence of rotational speed, screw and nozzle design, transition direction, and relative difference in material viscosity on material transitions printed with the BAAM dual-hopper was conducted. In particular, the relationship between the direction-sensitive nature of the transitions and material viscosities, which has been shown in recent studies to be greater than an order of magnitude [43], was expected to significantly impact transition behavior. Finally, a Weibull Cumulative Distribution Function (CDF) was applied to the raw data to obtain average transition curves. Through these analysis techniques, this study explores how to model material transitions and control transition length.

## 2. Materials and Methods

The influences of component design, print parameters, and relative difference in complex viscosity on transition behavior were investigated using the BAAM dual-hopper system to print material transitions at incremental screw speeds with different combinations of extrusion screw and nozzle designs. Each print utilizes a single-layer pattern to produce an independent transition for each material direction, i.e., material A to material B and B to A. These transitions were characterized using Ultrasonic Assisted Digestion (UAD) to track changes in material composition as a function of position in the deposited structure [39,42]. In turn, the extruded material volume was calculated from the travel distance (sample position), volumetric flow rate, and travel speed. The extruded volume was then normalized by the configuration-specific (extrusion screw and nozzle combination) residual volume of material, allowing for direct comparisons of material transition curves across print parameters and system configurations. Additionally, the standard Weibull CDF was chosen to fit experimental data and quantify transition comparisons and observations.

### 2.1. Materials and System Components

Three thermoplastic materials, two screw designs, and two nozzle designs were utilized for this study. ABS, 20 wt % carbon fiber-reinforced ABS (CFABS), and thermoplastic polyurethane (TPU) pelletized feedstocks were acquired under brand names HIFILL ABS 1512 3DP, ELECTRAFIL ABS 1501 3DP, and RESMART Ultra TPU 70A, respectively, from Techmer PM, Clinton, TN, USA. Two extrusion screws with identical feed and compression zone geometries were utilized in this study. While one had a uniform geometry in the metering zone (Standard), the other design utilized indentations on the shaft to encourage material blending (Mixing). Both screws were designed for a barrel 61.0 cm (24 in) long with diameter of 2.54 cm (1.0 in). Similarly, this study also investigated two nozzle designs: a standard hollow cylinder (Standard) and a proprietary design (Mixing) with alternating slotted obstructions intended to improve mixing within the polymer melt [44]. As a result, the Mixing variant was approximately 35 mm longer than the Standard version and had a 12.7 mm (0.5 inch) diameter compared to the 10.0 mm (0.4 inch) of the Standard nozzle. Changes in screw and nozzle design were made to investigate the impact of increased mixing during extrusion on overall transition behavior. Similarly, comparing an ABS-CFABS material pair to a TPU-CFABS material pair was intended to emphasize the relative difference in complex viscosities.

### 2.2. Sample Preparation

All samples were printed with the BAAM dual-hopper system using the same processing conditions for all parameters other than screw rotational speed and travel speed, which was programmatically adjusted using ORNL Slicer 2 from ORNL, Oak Ridge, TN, USA, by the slicing software as a function of screw speed. Software approximations also introduced minute differences in travel speed for identical screw speeds due to the different deposition patterns. As such, the exact travel speeds (*v*) are provided in Appendix A, Table A1. Four configurations, i.e., a combination of screw type, nozzle type, and material pair, were studied, and transitions were printed in both material directions, creating eight total transition categories. Table 1 provides these eight categories with associated identifiers. Each transition type was then printed at either six or three different screw rotational speeds, as indicated in Table 1. Thus, individual material transitions were identified by both an acronym and number, e.g., AX100 indicated an ABS to CFABS transition printed using the standard nozzle and standard screw at a rotational speed of 100 rotations per minute (RPM).

All other processing conditions were held constant. Using five heating zones on the barrel, a standard CF/ABS BAAM temperature profile was selected with a melt temperature of 250 °C. To maintain a stable melt temperature, the profile occasionally required slight adjustments during processing in response to changes in ambient conditions and system configuration. Prints using the Mixing nozzle required an additional heating sleeve held at 255 °C to prevent excessive cooling at the nozzle tip and maintain melt temperature. The deposition surface consisted of ABS build sheets held at 100 °C. Bead width and layer height were set to 1.4 cm (0.55 in) and 0.51 cm (0.2 in), respectively. All feedstock was dried at 80 °C for at least four hours in a vacuum-assisted hopper connected to the printer.

**Figure 1:**
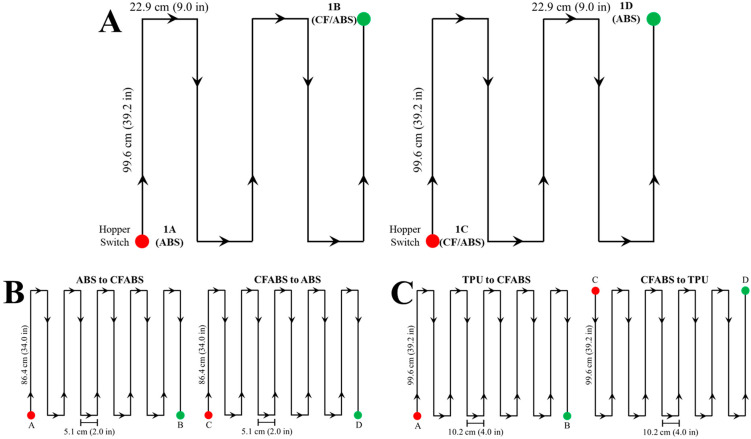
A schematic showing the three print paths used in this study: (**A**) The standard nozzle configuration; (**B**) The Mixing Nozzle configuration; (**C**) and The CFABS-TPU material pair configuration. Each print path utilized the same progression from Points A to B to C to D.

As shown by the schematic in Figure 1A, the toolpath defined two separate structures to simplify printing material transitions in both directions by separating them entirely. After purging until achieving steady-state ABS, the change in material is initiated by switching hoppers at 1A, as indicated by the red dot. The ABS to CFABS (1A to 1B) transition was then deposited in the specified serpentine pattern, reaching steady-state CFABS prior to 1B. Following a brief pause in extrusion for translation to 1C, the CFABS to ABS (1C to 1D) transition was deposited as shown using the same method. This ensured printed transitions had easily identifiable start points (red dots) that corresponded with the g-code command to switch materials. Two additional print paths with increased travel distance were required to complete this study due to changes in deposition behavior. Transitions printed with the mixing nozzle utilized the pattern shown in 1B to ensure steady-state material B occurred prior to the green dots (B and D). Similarly, visual observation of TPU-CFABS transitions suggested a longer blended region, so these prints utilized schematic 1C to ensure steady-state material B had been achieved prior to the green dots (B and D). The total lengths of a transition printed using patterns A, B, and C were 5.9 m, 9.1 m, and 10.9 m, respectively. As mentioned previously, the assigned bead dimensions and print parameters remained constant despite the changes in toolpath. After removal from the print bed, each transition structure was labeled to indicate cumulative travel distance (*d*) from the start point (red dot) and sectioned using a band saw for ease of storage and sample extraction. Using an Isomet 1000 precision saw from Buehler Ltd, Lake Bluff, IL, USA, samples approximately 10 mm long were taken from locations of interest. Previous work had demonstrated that a 10 mm sample size was consistently representative of the surrounding material composition [42]. Initial sample locations were chosen based on visual observation of color, and additional batches were selected based on the measured material compositions of previous batches to improve resolution of the transition curve. All extracted samples were dried for at least four hours at 80 °C prior to compositional analysis.

### 2.3. Method for Constituent Content Analysis

A custom method for obtaining the reinforcement content of a thermoplastic composite sample was developed and verified in previous work by consulting the procedures available in ASTM D3171-15 [39,42,45]. This technique, UAD, utilizes chemical dissolution rather than thermal decomposition to separate the constituent reinforcements from the matrix material, providing flexibility in analysis options through solvent selection. To avoid using highly reactive hydrogen peroxide as an accelerant, an ultrasonic water bath was incorporated to agitate the solutions and increase reaction speed. For this study, samples were submerged in 60 mL of Dimethyl Sulfoxide (DMSO) within a sealed, glass test tube and placed in an CPX 3800 Ultrasonic Bath heated to 50 °C for 10 h. DMSO was purchased from Chem-Impex, Wood Dale, IL, USA, and the CPX 3800 was purchased from Thermo Fisher Scientific, Waltham, MA, USA. DMSO was chosen as the solvent specifically to ensure dissolution of the TPU matrix [46]. A vacuum filtration system with Whatman Grade GF/A glass microfiber filters (47 mm diameter with 1.6 micron pores) from Grainger, Lake Forest, IL, USA, was used to separate fibers from the liquid solution. After drying overnight at 80 °C, the residual fiber mass was measured and divided by the original dry sample mass to determine fiber content by weight. Mass measurements were made using a RADWAG AS 220.R2 with 0.1 mg accuracy from RADWAG USA, Miami, FL, USA. Since CFABS was the only composite material, the measured fiber content was considered representative of material composition and used to track the progress of the material transition.

### 2.4. Density Measurements

Multiple material densities (*ρ*) were required to calculate volumetric flow rates (*Q*) and residual volumes (*V_R_*) for each of the three materials. Since the material state changes throughout the extrusion process, four different densities were identified: bulk density (*ρ_b_*) of the pelletized feedstock, the compressed bulk density (*ρ_c_*) of the feedstock, the melt density (*ρ_m_*), and the density of the extrudate (*ρ_E_*). The average bulk density, *ρ_b_*, for each feedstock was obtained from five measurements made using a custom apparatus constructed according to ASTM D1895 [47]. The compressed bulk densities, *ρ_c_*, were measured using a Dynisco Laboratory Capillary Rheometer 7001 (LCR) from Dynisco, Franklin, MA, USA, to apply pressure to a known mass of pellets in a chamber of known volume at room temperature. Changes in pressure during BAAM processing were considered negligible due to previous work demonstrating a minimal relationship between density and pressure at room temperature for amorphous thermoplastics [48]. Melt densities, *ρ_m_*, were found for each material at 250 °C using the LCR and following the procedure outlined in ASTM D3835 [49]. The density of extruded material, *ρ_E_*, was found using a Quantachrome Helium Pycnometer and standard deviation goal of 0.0005 g/cm^3^, consulting ASTM D792 and ASTM D6226 when possible [50,51]. The instrument was acquired prior to the acquisition of Quantachrome Instruments by Anton Paar GmbH.

### 2.5. Complex Viscosity Measurements

The viscoelastic properties of a material influence the selection of processing conditions for extrusion-based AM processes, so the complex viscosity, η*, of the three materials was measured to compare viscoelastic behavior under the chosen processing conditions. ABS, CFABS, and TPU samples were subjected to oscillatory frequency sweeps from 628 rad/s to 0.1 rad/s with an applied strain rate of 0.1% at 250 °C [43,52] using a Discovery Hybrid Rheometer-2 and 25 mm parallel plate geometry from TA Instruments, New Castle, DE, USA. The resulting η* values were compared to represent the difference in viscoelastic behavior at the chosen processing conditions. For TPU specimens, high-frequency values could not be obtained due to the relatively high processing temperature [53,54], so η* values were calculated using a power law estimation.

### 2.6. Volumetric Flow Rate Calculations

Volumetric flow rates (*Q*) were measured for each combination of material, screw speed, screw design, and nozzle design to enable accurate calculation of extruded volumes for all printed transitions. Per ASTM D1238, Procedure A [55], a thirty second extrusion at steady-state was conducted for each combination. *Q* was determined using the dry mass of the extrudate (*m*), elapsed time (*t*), and the extrudate density (*ρ_E_*), as shown in Equation (1).

(1) $$Q=\frac{m}{\rho_{E}\times t}$$

### 2.7. Comparing Material Transitions

Since the parameters studied directly influence the size and shape of the printed material, a comprehensive method for calculating and normalizing the cumulative volume of extruded material, *V_E_*, was developed. Normalizing *V_E_* by an associated residual volume of material in the system, *V_R_*, contextualized volume measurements by relating them to a relevant original volume and created an objective measurement system for plotting changes in material composition.

#### 2.7.1. Extruded and Residual Volume

**Figure 2:**
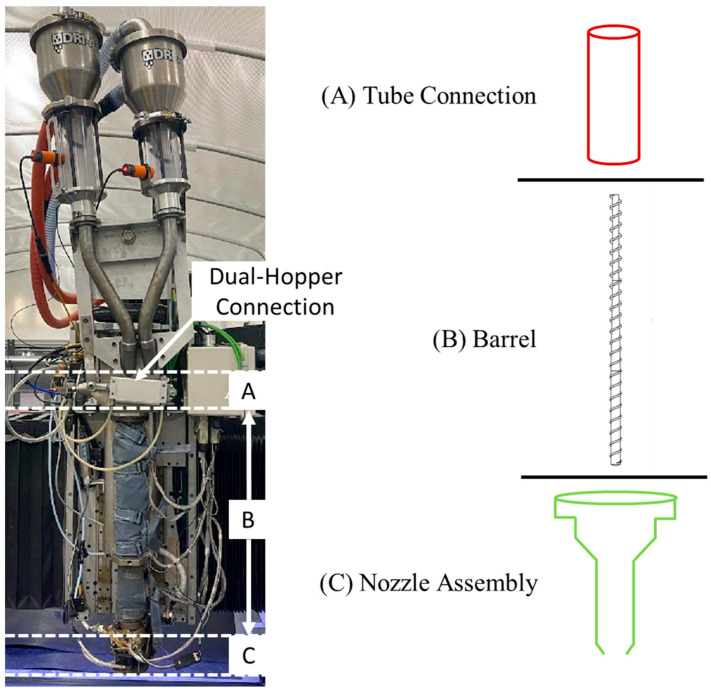
(**Left**) The BAAM dual-hopper extrusion head marked to show the three component regions of VR. (**Right**) Names and simple schematic representations of the (**A**) Feed Throat, (**B**) Barrel, and (**C**) Nozzle Assembly regions.

The process for calculating *V_E_* and *V_R_* was broken down into multiple steps due to differences in material characteristics and internal geometry of the system components, which is provided in detail in [56]. In the first, *V_E_* is calculated using *d*, *Q*, and *v*, accounting for any variations in bead morphology or extrudate behavior. The boundaries of *V_R_* were defined as starting at the material inlet and ending at the nozzle exit. This was then separated into three major regions as shown in Figure 2. Throughout *V_R_*, material was in a state other than extrudate, so a ratio of applicable density state (*ρ_b_*, *ρ_c_*, or *ρ_m_*) to *ρ_E_* was applied to convert the in-system material to an equivalent expected amount of extrudate. Thus, both *V_E_* and residual volume (*V_R_*) measurements were calculated in terms of extrudate volume while including differences in internal geometries for each configuration.

**Figure 3:**
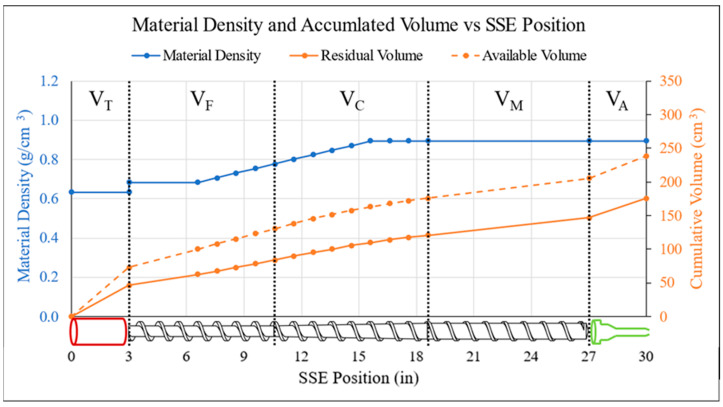
The change in material density plotted as a function of position in the BAAM (blue), and a comparison of available volume (dashed orange) to expected extrudate volume (solid orange) across the same region.

As can be seen in Figure 2A, the feed throat was approximated as a simple cylinder where the pelletized feedstock was not subjected to any additional heat or pressure as it traveled to the barrel entrance, so the chosen material density was simply *ρ_b_*. Although the barrel itself was also a cylinder, the embedded extrusion screw prohibited any straightforward calculations. Instead, this region was approximated by subtracting the volume of the associated digital model from a cylinder with dimensions equivalent to the barrel. Since the feed material undergoes melting in this region, a location-specific blend of *ρ_c_* and *ρ_m_* was applied to each screw flight using a stepwise gradient and linear rule of mixtures. For the nozzle assembly region, the internal free volume was obtained from the digital models and multiplied by the melt density ratio to represent the uniform material state. A representation of this approach is displayed in Figure 3 to illustrate how the accumulation of available material diverges from the available space inside the assembly as a function of material state.

After establishing a standard approach for calculating extruded (*V_E_*) and residual (*V_R_*) volume, additional steps were taken to include differences in material properties and the continuously changing composition in the blended material region. Equation (2)

(2) $$Q_{R}=Q_{A}\left(F_{A}\right)+Q_{B}\left(1-F_{A}\right)$$

averages the material-dependent *Q* using a linear Rule of Mixtures (ROM) and measured material composition (*F_A_* and *F_B_*), which leads to the final formula for determining *V_E_* in Equation (3):

(3) $$V_{E}=V_{i}+\frac{Q_{R}(d_{i}-d_{i-1})}{v}$$

This clarifies how travel distance was used in [56] by introducing *V_i_* and *d_i_* to ensure that the calculated ROM volumetric flow rate (*Q_R_*) was not retroactively applied to the entire travel distance. Here, *V_i_* represents the *V_E_* calculated for the most recent sample measurement and *d_i_* is the distance traveled up to the current sample. Table A2 lists the flow rates used to find *Q_R_*.

Similarly, the material dependency of bulk density (*ρ_b_*), compressed bulk density (*ρ_c_*), melt density (*ρ_m_*), and extrudate density (*ρ_E_*) affected the calculations for *V_R_*, so a distinct residual volume was calculated for each of the three materials. To estimate the effect of the blended material properties, a blended residual volume was also calculated for each measured material composition using Equation (4), where *V_R_*_−*A*_, *V_R_*_−*B*_, and *V_Rx_* represented the material A, material B, and rule of mixtures residual volumes, respectively.

(4) $$V_{Rx}=V_{R-A}\left(F_{A}\right)+V_{R-B}\left(1-F_{A}\right)$$

#### 2.7.2. Transition Curve Construction and Analysis

To analyze material transition behavior, transition curves were constructed by plotting material composition as a function of normalized volume, *V_N_*. Mathematically, *V_N_* represented the ratio of extruded volume (*V_E_*) to residual volume (*V_R_*), as shown in Equation (5).

(5) $$V_{N}=\frac{V_{E}}{V_{Rx}}$$

More importantly, this approach removed the influence of external factors unrelated to fundamental transition behavior, enabling comparisons across print conditions and configurations. Thus, the characteristics of individual transition curves could be compared to identify changes in mixing and transition behavior while excluding any inherent differences caused by processing conditions.

**Figure 4:**
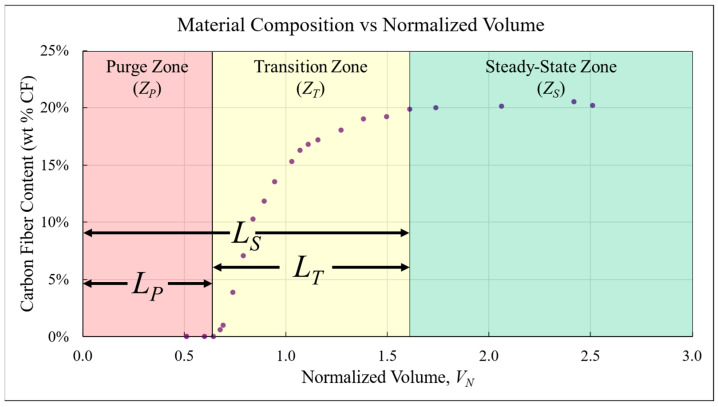
A color-coded diagram showing the three regions of a material transition curve.

As reported in previous work [42,57,58], BAAM dual-hopper transition curves consistently displayed three characteristic zones: Purge (*Z_P_*), Transition (*Z_T_*), and Steady-State (*Z_S_*). Figure 4 uses an example data set to visualize the three zones and establish measurable characteristics of a transition. The length of the purge zone (*L_P_*) represents the volume of material required for material composition to begin changing while the length required to complete a transition (*L_S_*) represents the material volume required to achieve steady-state printing of the second material. The length of the transition zone (*L_T_*) was found using Equation (6):

(6) $$L_{T}=L_{S}-L_{P}$$

The influence of a parameter was assessed by comparing these lengths *L_P_*, *L_T_*, and *L_S_*.

#### 2.7.3. Modeling Techniques

Although transition curves constructed from compositional measurements provided significant insight into transition behavior, the slight noise associated with UAD [42,58] limited definitive results. Fitting the experimental data to existing models presented a possible means for refining qualitative results, so transitions were plotted using percent Material B rather than wt % CF to provide a simple 0 to 100 boundary condition. First, a custom function was utilized to create a piecewise model of the entire transition curve, as described in [56]. After extracting *L_P_* from this function, a Weibull CDF, shown in Equation (7),

(7) $$f\left(x\right)=1-{exp}^{{\left(\frac{-x}{\lambda }\right)}^{k}},$$

was applied as if *L_P_* were zero to find best fit values for *λ* and *k* based on experimental data in the transition and steady-state zones.

**Figure 5:**
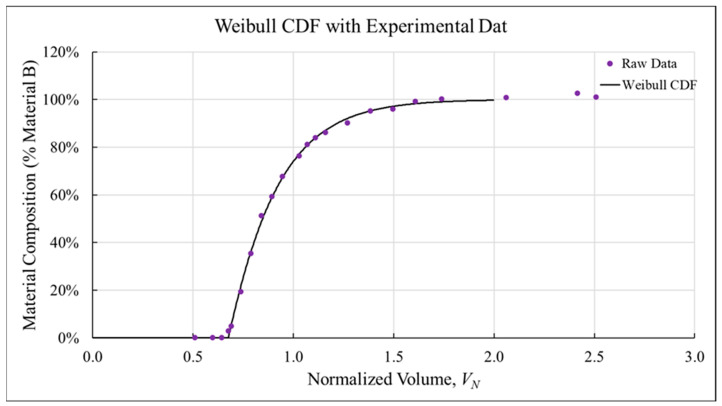
An example of the Weibull CDF applied to the data set shown in Figure 4.

Effectively, this created a delayed Weibull CDF where the start of the CDF was identified mathematically instead of choosing from experimental data points, improving on the fits obtained in previous work [56]. The best fit values for the Weibull CDF parameters (*λ* and *k*) were found using a MATLAB (MATLAB R2023a from MathWorks, Natick, MA, USA) script to apply the CDF to each set of raw data by minimizing residuals using nonlinear least square regression. The point of transition completion, i.e., *L_S_*, was identified as the point where the associated Weibull CDF was equal to 0.99. In some cases, experimental transition curves exhibited composition thresholds below the expected 20 wt % carbon fiber content, so the threshold for determining *L_S_* was adjusted from 0.99 to 0.95. This enables comparisons between the relative lengths of material transitions while also providing a secondary analysis tool through comparisons of *λ* and *k*. As an example, Figure 5 shows the original raw data points with the associated Weibull CDF overlaid as a solid line.

#### 2.7.4. Curve-Shifting to *V*_1_

**Figure 6:**
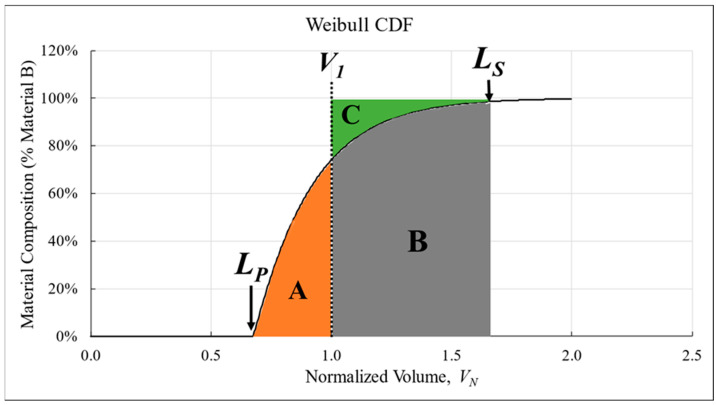
Graphical depiction of the areas described by Equation (8). Regions A and C represent the equivalent volumes created by the mixing process where region B is used to find A and C.

To ensure the model agreed with the physical reality of the BAAM dual-hopper, a post-processing step was included after fitting data to the Weibull CDF. Based on conservation of mass, the finite material volume, *V_R_*, should be reflected in the associated transition curves. Using a boundary condition of *V_N_* equal to 1 (*V*_1_) where an instantaneous change in material would occur, Figure 6 shows graphically how the area under the curve prior to *V*_1_ (A), the area between a transition curve and 100% Material B following *V*_1_ (C), and the area under the curve following *V*_1_ (B) were constrained. This graphical analysis led to a mathematical solution for finding areas A and C, as shown in Equation (8), where *F*(*x*) represents the Weibull CDF.

(8) $$\int_{L_{P}}^{V_{1}}F\left(x\right)dx=C=\int_{V_{1}}^{L_{S}}1dx-\int_{V_{1}}^{L_{S}}F\left(x\right)dx$$

Following these principles, all transition curves exhibited different values for areas A and C. Therefore, the final adjustment made to the modeled transition curves was a translation of the curve on the *x*-axis until the areas were of equal size using a MATLAB script that incrementally shifted both *L_T_* and *L_S_* simultaneously until Areas A and C were within 0.1% of each other. This preserved the shape and parameter values of the Weibull CDF while correcting for any error introduced by inconsistent processing conditions or environmental factors. The values by which each curve was shifted can be found in Appendix A, Table A3.

## 3. Results and Discussion

### 3.1. Material Properties

Density measurements were obtained for the ABS, CFABS, and TPU feedstocks while in four different material states. Table 2 contains *ρ_b_*, *ρ_c_*, and *ρ_m_* for each material. There was a negligible increase from bulk to compressed bulk density for ABS and TPU, which agreed with literature [48]. Although CFABS exhibited a 5% increase, this was attributed to breakage or deformation of the constituent fibers and the higher void fraction associated with composite thermoplastics allowing the pellets to compress rather than compression of the ABS matrix. ABS exhibited the highest melt density while TPU was both the least dense and experienced the smallest increase from *ρ_b_*. ABS and CFABS exhibited similar densification, increasing by 38% and 41% from *ρ_b_*, respectively. Initial extrudate density (*ρ_E_*) measurements revealed minor fluctuations in density with processing conditions, so each transition curve utilized a unique *ρ_E_*. These values are provided in Table A2 along with *Q* for reference.

As a part of this work, the complex viscosities (η*) of each material were experimentally measured to quantify their expected viscoelastic behavior at BAAM processing conditions. Results indicated that CFABS would be approximately an order of magnitude more viscous than ABS while TPU would be multiple orders of magnitude less viscous than both ABS and CFABS. The experimental details can be found in [56].

### 3.2. Normalized Volume

**Figure 7:**
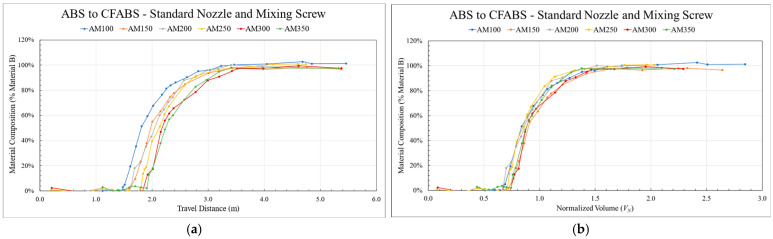
Two charts showing the same data sets plotted as (**a**) a function of travel distance from the point of hopper switch and (**b**) a function of extruded volume normalized by the residual volume.

As discussed in Section 2.7, transition curves were assessed using a novel normalized volume approach. Figure 7 demonstrates the importance of this method. As can be seen in Figure 7a, assessing the transitions as a function of travel distance would suggest a significant difference in behavior. However, implementing the normalized volume approach caused the curves to collapse into similar shapes, as shown in Figure 7b. This suggested the existence of an average curve for this system configuration independent of screw speed. As intended, these results further justified using the described normalization technique to analyze fundamental transition behavior and compare different processing conditions. Furthermore, data obtained under similar conditions could also be reverse engineered to predict where blended regions would occur when printed using different bead geometries or screw speeds, aiding specialized part design and intentional placement.

### 3.3. Weibull CDF Fit

**Figure 8:**
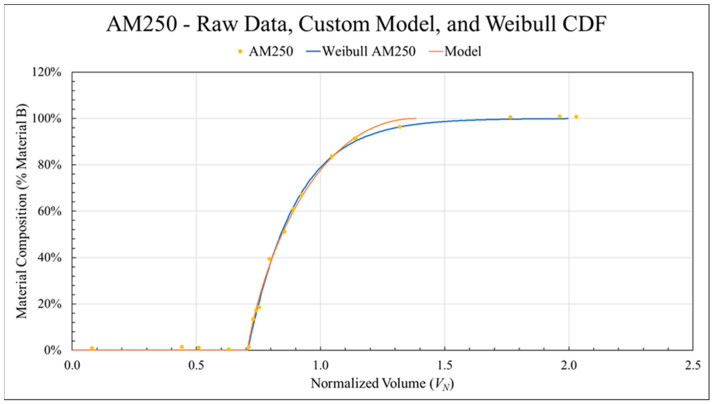
An example data set showing the improvement in fit offered by the Weibull CDF compared to the originally developed custom model.

In previous work [56], a custom curve-fitting approach utilizing least squares regression was implemented to model the experimental transition curves so that the transition zone boundaries were more clearly defined. This technique was again utilized to find the starting point of each transition, *L_P_*, but a Weibull CDF was used to model the progression from *L_P_* to *L_S_* by treating the *L_P_* boundary point as x = 0. Figure 8 compares the experimental data, the custom model fit, and the Weibull CDF fit for the AM250 (ABS to CFABS, standard nozzle, mixing screw at 250 RPM) data set. The difference in *L_S_* can be seen visually and represented a 12% increase in transition length change from 1.386 to 1.552 residual volumes. As such, the Weibull CDF provides much needed improvement to the modeling process.

**Figure 9:**
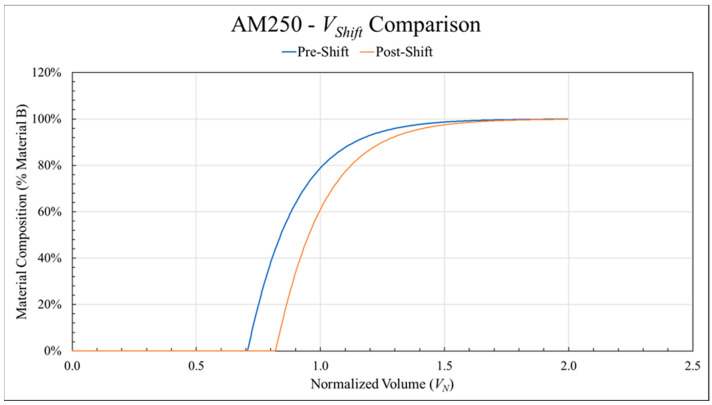
The AM250 data set before (blue) and after (orange) applying the *V_Shift_* correction factor to preserve conservation of mass.

In addition to normalization, a secondary adjustment was made to transition curves as part of the analysis and modeling to conform to conservation of matter principles. After determining *λ* and *k*, *L_S_* was found as described in Section 2.7.3. Then, both *L_P_* and *L_S_* were translated on the *x*-axis by the associated *V_Shift_* (Table A2) as described in Section 2.7.4, preserving the shape of the Weibull CDF fit without altering *λ* and *k*. Figure 9 illustrates the effect by showing the AM250 data set used by Figure 8 before (blue) and after (green) applying the *V_Shift_* translation. Once again, the transition length increased 7% from 1.552 to 1.665, demonstrating the importance of this step. This final adjustment ensured the modeled transitions were adhered to physical limitations.

### 3.4. Assessing the Effect of Screw Speed

**Figure 10:**
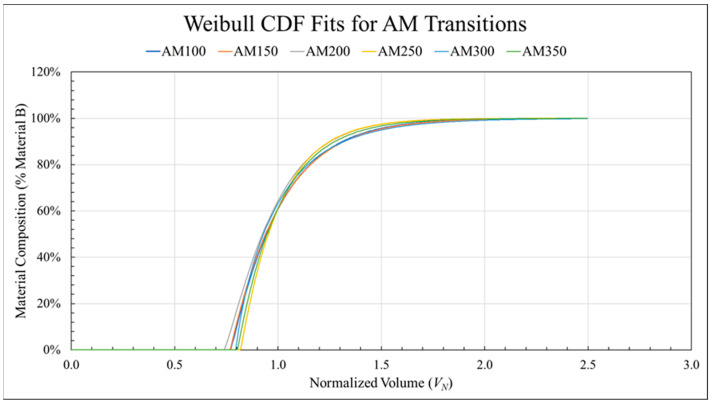
The Weibull CDF fit for all six material transitions printed in the ABS to CFABS direction using the mixing screw and standard nozzle.

Having found *L_P_*, *L_S_*, *k*, and *λ* for all printed transitions, the AM (ABS to CFABS, mixing screw, standard nozzle) configuration was used to investigate the effect of screw rotational speed on transition behavior. All six transition curves are shown in Figure 10 and labeled with the associated screw speed. Changes in screw speed did not lead to any trend in transition behavior as judged by *L_P_* and *L_S_*. The standard deviation in the purge length (*L_P_*) was less than 0.03 *V_R_* while the standard deviation in overall transition length was 0.11 *V_R_*. Although there was no clear trend in *λ*, there did appear to be two groupings where the three lower screw speeds were near 0.25 and the three higher screw speeds were near 0.20. Additionally, *k* consistently decreased with increasing screw speed. Similar behavior was seen in the remaining configurations. Since there were no strong trends observed as a function of screw speed, transitions printed using the same configuration were condensed into an average curve by averaging the four variables *L_P_*, *L_S_*, *k*, and *λ* for ease of comparative analysis.

### 3.5. Effect of Transition Direction

**Figure 11:**
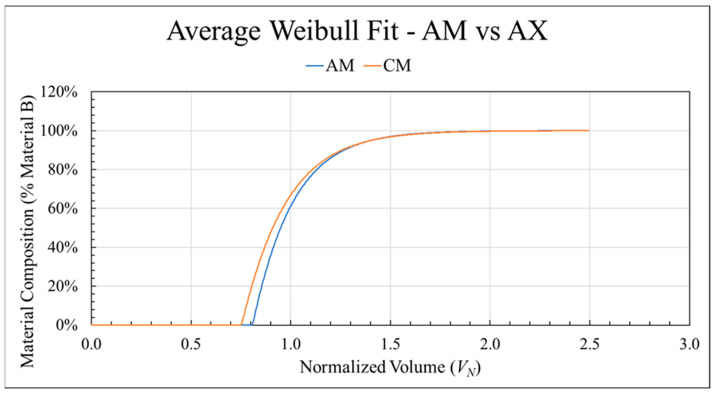
The average transition curves for both sample sets printed using the mixing screw and standard nozzle: ABS to CFABS (blue) and CFABS to ABS (orange) directions.

The effect of transition direction was analyzed by comparing transitions printed using the same configuration but in opposite directions, i.e., A to B vs. B to A. As mentioned, the CM (CFABS to ABS, mixing screw, standard nozzle) configuration exhibited the same lack of response to changes in screw speed. To directly compare transition directions, Figure 11 shows the average curves for both the AM and CM configurations. The behavior diverges immediately as *L_P_* decreased by 6.9% (0.808 to 0.752) when switching the starting material from ABS to CFABS. Similarly, the overall transition length was 5.0% shorter in the CFABS to ABS direction at 1.632 compared to 1.718. However, the length of the blended transition zone, *L_T_*, was comparable (0.91 and 0.88). In addition, *k* values were nearly identical (1.02 and 1.003), but *λ* increased significantly from 0.203 to 0.294 (44.8%). Since all other factors remained constant, the decreased transition lengths were attributed to the difference in material properties, specifically complex viscosity. Here, transitioning from high to low viscosity reduced both the initial purge zone and the overall transition length.

### 3.6. Effect of Screw Design

**Figure 12:**
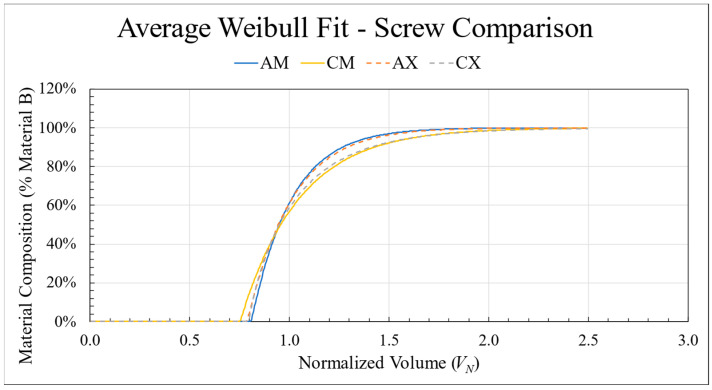
The average curve fits for two configurations in both directions: mixing screw with standard nozzle (solid lines) and standard screw with standard nozzle (dashed lines).

The first change in configuration switched the customized mixing screw for a standard SSE screw. Using the average curve for each configuration, the AM, CM, AX, and CX curves were plotted in Figure 12 to illustrate the effect of screw geometry. Transitions using the mixing screw were given solid lines while the standard screws used dashed lines. While the standard screw elongated the overall transition for the ABS to CFABS direction (1.72 to 1.79), the CFABS to ABS direction had similar lengths. Instead, the standard screw increased the length of the purge zone, leading to a comparatively shorter blended transition zone. Although the change in screw design had a small influence on transition behavior, the most influential factor remained the difference in materials.

### 3.7. Effect of Nozzle Design

**Figure 13:**
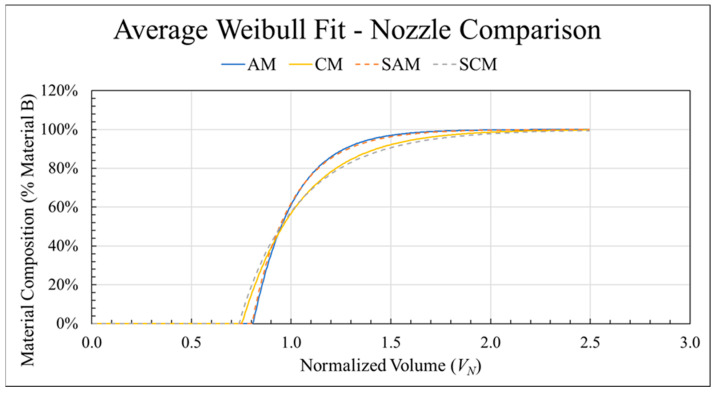
The average curve fits for the standard (solid) and mixing (dashed) nozzles.

The effect of nozzle design was investigated using only the mixing screw, so the AM and CM configurations were compared to the SAM (ABS to CFABS, mixing screw, mixing nozzle) and SCM (CFABS to ABS, mixing screw, mixing nozzle) using average curve values, as shown in Figure 13 where standard nozzles were indicated by solid lines and the mixing by dashed lines. The influence of the mixing nozzle was much clearer than the screw. In both instances, there was a negligible difference in purge zone length (*L_P_*), but the mixing nozzle increased the length of the transition zone (*L_T_*) by roughly 0.09 *V_R_*, leading to longer overall transitions. This development was attributed to the significant decrease in volumetric flow rates (Table A2) seen while using the mixing nozzle. With all other conditions held constant, this would have caused an increase in backpressure flow throughout the SSE system. In turn, this would be expected to increase the interaction between the competing pressure and drag flows, resulting in material mixing earlier in the extrusion process. For dual-hopper material transitions, this would most likely produce longer transition zones like those seen here.

### 3.8. Effect of Relative Complex Viscosity

**Figure 14:**
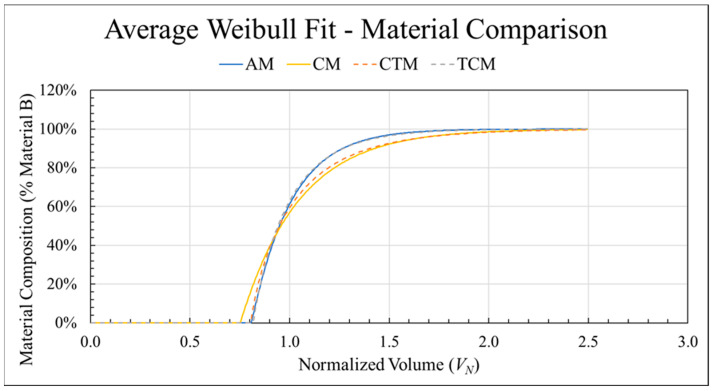
The average transition curves for mixing screw with standard nozzle configuration in both directions for both the ABS-CFASB (solid) and TPU-CFABS (dashed) material pairs.

Given the importance of complex viscosity to drag and pressure flow in extrusion processes, the relative difference in η* was expected to significantly impact transition behavior. Figure 14 compares the average AM and CM transition to the TCM (TPU-CFABS, mixing screw, standard nozzle) and CTM (CFABS to TPU, mixing screw, standard nozzle) configurations. When starting with CFABS, the switch to TPU significantly shortened the transition zone length but not the overall length by increasing the purge zone length. In the opposite direction, the transition zone and overall transition length both increased while the purge zone length remained the same. This indicated that a greater difference in complex viscosity impacted transition behavior differently based on the direction of the material change. When comparing the TCM and CTM curves, *L_S_* decreased significantly when transitioning from high to low (CTM) than low to high (TCM). As expected, these results suggested that the mixing behavior observed in material transitions was both design and property dependent. The transition boundaries and Weibull parameters for all configurations are summarized in Table 3, below.

## 4. Conclusions

Numerous material transitions printed using the BAAM dual-hopper system were analyzed to assess the influence of material properties and system components on transition behavior. To objectively compare material transitions printed under different circumstances, a novel framework was developed based on material properties and system constants. After building this framework, each material transition was analyzed by plotting material composition as a function of normalized volume and fitting the curves using a Weibull CDF model. Through these steps, the characteristic start, stop, and blended zone lengths were compared across system components and print parameters.

The effect of screw speed on zone lengths was either inconclusive or minimal such that there were no trends related to increasing or decreasing rotational speeds. Therefore, the zone lengths *L_P_*, *L_S_*, and *L_T_* and Weibull CDF parameters *k* and *λ* were averaged to obtain an average representative curve for each configuration. Introducing additional mixing components increased the lengths of the purge zone (*L_P_*) when transitioning from low viscosity to high but decreased *L_P_* when transitioning from high to low viscosity materials. This effect was attributed to the increased back pressure flow caused by the mixing designs leading to material mixing sooner during the extrusion process. Similarly, the length of the blended zone (*L_T_*) and overall transition (*L_S_*) increased when using mixing components. Both *k* and *λ* were similar to *L_P_* in that behavior differed depending on transition direction. For low to high transitions, both parameters decreased with the inclusion of mixing components. For high to low transitions, both parameters increased with the addition of mixing components.

These observations indicate that the continuous material transitions printed using the BAAM dual-hopper can be influenced by intentional design of components and material choice. By controlling the relative difference in material viscosity, the characteristics of a material transition can be adjusted for intentional design and site-specific properties. With these capabilities, printed parts and print paths can be designed to reduce surface contact between dissimilar materials, increasing z-direction strength through improved bonding and reduced delamination. This study also demonstrates that a Weibull CDF can be used as a model for transition behavior. As shown by the significant change in value when switching screw and nozzle designs, the Weibull parameters represent the impact of the system configuration on mixing behavior. Further study of this relationship could develop systematic design criteria for components using the Weibull parameters. In addition to this possibility, future investigations should utilize temperature and molecular weight to influence transition behavior through changes in complex viscosity.

## Figures and Tables

**Table 1:** From left to right, a summary showing how component and material pairings were grouped into four configurations and then eight categories based on transition direction.

Screw Geometry	Nozzle Geometry	Material Direction	Identifier	Screw Speed (RPM)					
Standard	Standard	ABS to CFABS	AX	100	150	200	250	300	350
		CFABS to ABS	CX	100	150	200	250	300	350
Mixing	Mixing	ABS to CFABS	AM	100	150	200	250	300	350
		CFABS to ABS	CM	100	150	200	250	300	350
Standard	Standard	ABS to CFABS	SAM	100	--	200	--	300	--
		CFABS to ABS	SCM	100	--	200	--	300	--
Mixing	Mixing	TPU to CFABS	TCM	100	--	200	--	300	--
		CFABS to TPU	CTM	100	--	200	--	300	--

**Table 2:** The average bulk, compressed bulk, and melt densities of the three studied materials.

Material	Bulk Density, *ρ_b_* (g/cm^3^)	Compressed Bulk Density, *ρ_c_* (g/cm^3^)	Melt Density, *ρ_m_* (g/cm^3^)
ABS	0.664	0.653	0.916
CFABS	0.633	0.6683	0.894
TPU	0.715	0.715	0.876

**Table 3:** The transition characteristics for all studied configurations and material pairings.

Transition Direction	ABS to CFABS			TPU to CFABS	CFABS to ABS			CFABS to TPU
Screw Design	Stan	Mix	Mix	Mix	Stan	Mix	Mix	Mix
Nozzle Design	Stan	Stan	Mix	Stan	Stan	Stan	Mix	Stan
*L_P_*, Purge Length	0.788	0.808	0.804	0.818	0.798	0.752	0.740	0.806
*L_S_*, Overall	1.789	1.718	1.804	1.810	1.631	1.632	1.716	1.629
*L_T_*, Transition	0.999	0.910	1.000	0.992	0.834	0.879	0.976	0.823
*k*	1.029	1.02	0.963	0.926	0.862	1.004	0.947	0.833
*λ*	0.224	0.203	0.202	0.184	0.233	0.294	0.306	0.220

## Data Availability

The original contributions presented in this study are included in the article. Further inquiries can be directed to the corresponding author.

## Abbreviations

The following abbreviations are used in this manuscript:

ABS	Acrylonitrile Butadiene Styrene
AM	Additive Manufacturing
BAAM	Big Area Additive Manufacturing
CDF	Cumulative Distribution Function
CFABS	Carbon Fiber-Reinforced ABS
DMSO	Dimethyl Sulfoxide
FGM	Functionally Graded Material
LCR	Laboratory Capillary Rheometer
LFAM	Large Format Additive Manufacturing
MM	Multiple Materials
MMAM	Multi-Material Additive Manufacturing
ROM	Rule of Mixtures
RPM	Rotations Per Minute
TPU	Thermoplastic Polyurethane
UAD	Ultrasonic Assisted Digestion

## Appendix A

**Table A1:** Travel speeds, *v*, for each material transition.

Configurations	RPM	Travel Speed, *v* (m/s)
ABS to CFABS using standard screw and standard nozzle (AX) and mixing screw and standard nozzle (AM); CFABS to ABS using standard screw and standard nozzle (CX) and mixing screw and standard nozzle (CM)	100	0.036
	150	0.054
	200	0.073
	250	0.091
	300	0.109
	350	0.127
TPU to CFABS using mixing screw and standard nozzle (TCM); CFABS to TPU using mixing screw and standard nozzle	100	0.036
	200	0.072
	300	0.109
ABS to CFABS using mixing screw and mixing nozzle (SAM); CFABS to ABS using mixing screw and mixing nozzle (SCM)	100	0.038
	200	0.076
	300	0.114

**Table A2:** Volumetric flow rates and calculated densities for each material transition.

Description	Nozzle Design	Density, *ρ_E_* (g/cm^3^)	Measured *Q* (×10^−6^ m^3^/s)
ABS at 100 RPM	Standard	1.0538	2.9
ABS at 150 RPM		1.0492	4.3
ABS at 200 RPM		1.0446	5.4
ABS at 250 RPM		1.0482	6.4
ABS at 300 RPM		1.0518	7.7
ABS at 350 RPM		1.0500	8.7
CFABS at 100 RPM		1.1245	3.2
CFABS at 150 RPM		1.1134	4.6
CFABS at 200 RPM		1.1024	5.9
CFABS at 250 RPM		1.1020	6.8
CFABS at 300 RPM		1.1016	8.1
CFABS at 350 RPM		1.1018	9.2
ABS at 100 RPM	Mixing	1.0356	2.9
ABS at 200 RPM		1.0356	5.1
ABS at 300 RPM		1.0356	6.6
CFABS at 100 RPM		1.0918	2.8
CFABS at 200 RPM		1.0918	4.9
CFABS at 300 RPM		1.0918	6.5
TPU at 100 RPM	Standard	1.1844	3.5
TPU at 200 RPM		1.1860	7.8
TPU at 300 RPM		1.1829	10.1

**Table A3:** The amount of x-direction translation required for each curve, in terms of *V_R_*.

Configuration	100 RPM	150 RPM	200 RPM	250 RPM	300 RPM	350 RPM
AM	0.0957	0.0263	0.1006	0.1132	0.0481	0.0648
AX	0.0785	0.1016	0.1146	0.1659	0.1058	0.1692
CM	−0.0536	−0.1347	−0.1353	−0.0787	−0.0555	−0.0079
CX	−0.0989	−0.0888	−0.0151	0.0109	0.0319	0.0649
SAM	−0.1208		−0.0343		−0.0054	
SCM	−0.1208		−0.0103		0.0279	
CTM	−0.0926		−0.0959		0.0034	
TCM	−0.0077		0.0080		0.0409	
